# Effect of subconjuctival and intraocular bevacizumab injection on angiogenic gene expression levels in a mouse model of corneal neovascularization

**Published:** 2009-11-13

**Authors:** Olga Dratviman-Storobinsky, Bat-Chen R. Avraham Lubin, Murat Hasanreisoglu, Nitza Goldenberg-Cohen

**Affiliations:** 1 The Krieger Eye Research Laboratory, Felsenstein Medical Research Center, Petah Tiqwa, Israel; 2 The Mina and Everard Goodman Faculty of Life Sciences, Bar Ilan University, Ramat Gan, Israel; 3 Department of Ophthalmology, Pediatric Division of Ophthalmology, Schneider Children’s Medical Center of Israel, Petah Tiqwa, Israel; 4 Sackler School of Medicine, Tel Aviv University, Tel Aviv, Israel

## Abstract

**Purpose:**

This study sought to characterize the expression of angiogenesis-related genes in a mouse model of corneal neovascularization, either untreated or after treatment with a single injection of bevacizumab by three different routes. In addition, the effectiveness of the treatment was compared to a rabbit model.

**Methods:**

A chemical burn was induced in the mid-cornea of the right eye in 119 mice; 56 of them were untreated and 63 were bevacizumab-treated. Neovascularization was evaluated 2, 4, 8, 10, and 14 days later using digital photos, angiography and India ink perfusion. The relative area of new blood vessels was analyzed using slit-lamp examination in vivo and on histological and flat-mount sections. The levels of gene expression involved in the angiogenic process vascular endothelial growth factor [VEGF], insulin-like growth factor-1 [IGF-1], pigment epithelium derived factor [PEDF], and macrophage-inflammatory protein-2 [MIP-2]) were measured by a real-time polymerase chain reaction. Six rabbits underwent the same injury and treatment, and the response was compared to the mouse model.

**Results:**

Neovascularization was first observed two days after injury. The affected section increased from 11.24% (±7.0) of the corneal area to 47.42% (±25.4) on day 8 and 50.62% (±24.7) on day 10. In the mice treated with bevacizumab, the relative area of neovascularization was significantly lower at the peak time points (p<0.005): 24.90% (±21.8) on day 8 and 28.29% (±20.9) on day 10. Spontaneous regression was observed on day 14 in both groups, to 26.98% (±19.9) in the untreated mice and 10.97% (±10.8) in the bevacizumab-treated mice (p<0.005). Rabbits also showed peak corneal neovascularization on days 8-10, with significant regression of the vessels following intracameral bevacizumab injection. In the mice, intraocular (intravitreal, intracameral) injection was more effective than subconjuctival injection. *VEGF* gene expression was upregulated in both the untreated and treated mice, but was slightly less in the treated mice. *PEDF* gene expression decreased in both the treated and untreated mice. In the untreated group, gene expression peaked (above baseline) at 14 days, and in the untreated mice, it had already peaked by day 8. IGF-1 was upregulated early in the model; at 8 days, there was only a slight change in the untreated group compared to a significant increase in the treated group. MIP-2 was upregulated in both groups in the early stage and returned to baseline on day 14.

**Conclusions:**

Bevacizumab treatment partially inhibits the progressive corneal neovascularization induced by chemical damage in a mouse model. Treatment is more effective when administered via the intraocular than the subconjunctival route. The clinical findings are compatible with the angiographic and histologic data and are supported by molecular analysis showing a partial change in expression of proangiogenic genes. The molecular mechanisms involved in corneal neovascularization and inflammation warrant further exploration. These findings may have important therapeutic implications in the clinical setting.

## Introduction

Angiogenesis is one of the key factors in tumor progression and metastasis. Anti-angiogenic therapy inhibits tumor angiogenesis and promotes apoptosis of existing blood vessels, thereby intercepting the tumor's supply of oxygen and nutrients. Furthermore, by normalizing vascular permeability, anti-angiogenic therapy can improve the delivery of therapeutic agents to tumor cells.

A normal, healthy cornea is devoid of both blood and lymphatic vessels [1-3], but inflammatory conditions such as chemical burns, trauma or infection can cause angiogenesis [4-6]. The development of new blood vessels in the cornea, called neovascularization, is a final pathway common to all insults that are resistant to treatment and may lead to severe impairment of vision. Corneal neovascularization has been reported in 4.14% of patients presenting for general ophthalmologic care in the USA, representing an estimated 1.4 million individuals [7].

Until recently, the mainstay of anti-angiogenic therapy in the cornea was nonspecific anti-inflammatory drugs and sometimes anti-angiogenic steroids, but they were often unable to prevent or stop neovascularization [8]. Even surgical treatment with corneal transplantation frequently failed, as the new vessels induced an inflammatory rejection of the graft [7,9]. However, the rapid progress in angiogenesis research in the last few years has led to the development of several novel, specific anti-angiogenic drugs for use in both oncology and ophthalmology [10]. As the vast majority of these agents are low-molecular-weight compounds with a poor pharmacokinetic profile and rapid clearance rate, researchers have found that by conjugating them to polymeric carriers, their solubility and specificity may be increased and their pharmacokinetics is improved [11,12]. Recent reports describe the development of polymeric nanospheres that selectively target the activated vascular endothelium and can deliver encapsulated anti-angiogenic agents in cases of inflammatory disease with an angiogenic component [13].

A major focus of the research into anti-angiogenic therapy is vascular endothelial growth factor (VEGF), which is known to promote several steps in angiogenesis, including proteolytic activities, endothelial cell proliferation, endothelial cell migration and capillary tube formation [6]. Its essential role in normal embryonic vasculogenesis and angiogenesis was supported by findings that inactivation of a single *VEGF* allele in mice resulted in death of the embryo [14]. VEGF exerts its activity by binding to several high-affinity transmembrane endothelial cell receptors, especially VEGFR-1 (Flt-1) and VEGFR-2 (KDR/Flk-1). This leads to intracellular receptor phosphorylation, which in turn triggers the relevant intracellular downstream receptor pathways [15]. VEGF has been found to play a role in corneal neovascularization in both experimental models and humans [16-18]. Measurements of VEGF molecules and receptors in diseased corneas yielded higher concentrations than in normal or avascular abnormal corneas. Treatment strategies targeting the VEGF signaling pathway include anti-VEGF antibodies, soluble receptors binding directly to the VEGF ligand, anti-VEGF receptor (VEGFR) antibodies and VEGFR tyrosine kinase inhibitors. Anti-VEGF therapy has proven highly effective in treating advanced colon cancer and is currently being tested in gastric cancer [19]. In several studies, the manipulation of various anti-VEGF agents led to effective inhibition of corneal neovascularization [3,20].

Bevacizumab (Avastin; Genentech, San Francisco, CA) is a full-length recombinant humanized murine monoclonal antibody that binds to and inhibits the biological activity of all five human VEGF-A isoforms: VEGF115, VEGF121, VEGF165, VEGF189, and VEGF206 [21]. It prevents VEGF-A from ligating to its endothelial receptors, VEGFR-1 and VEGFR-2 [22,23], but does not neutralize other members of the VEGF gene family, such as VEGF-B or VEGF-C [24-26]. The antibody was engineered by assembling VEGF-A binding residues from the murine-neutralizing antibody into the framework of a human immunoglobulin [27]. Bevacizumab has been approved by the US Food and Drug Administration for use in the treatment of metastatic colorectal cancer. Since mid-2005, it has been applied off-label in the treatment of ocular disease and has shown promising short-term results in alleviating intraocular neovascular conditions [28-30].

Reports that the systemic application of bevacizumab in animal models inhibited inflammatory corneal neovascularization [20,23] led to the assumption that bevacizumab might also be clinically beneficial in patients who do not respond to conventional steroid therapy. This was followed by successful experiences with bevacizumab applied as eye drops [31-33] or subconjunctivally [34-36] in patients with progressive corneal neovascularization. Topical treatment was found to be safe and efficient, without local or systemic adverse effects [37,38]; some authors suggested that it could serve as a pre-transplantation treatment option [39]. Further studies reported a reduction in corneal inflammation and choroidal neovascularization after subconjunctival injection of bevacizumab [35,40-43], and a reduction in neovascular glaucoma after intracameral injection [44,45]. Intravitreal injection is currently the most common route of treatment for age-related macular degeneration with choroidal neovascularization [46].

Studies have shown that there is a balance in the cornea between angiogenic molecules such as VEGF and erythropoietin (EPO) and anti-angiogenic molecules such as pigment epithelium derived factor (PEDF). Angiogenesis and erythropoiesis represent adaptive responses to hypoxia and are upregulated in ischemic conditions. PEDF, a 50-kDa protein secreted by the retinal pigment epithelium, inhibits the growth of new blood vessels, most likely via the PI3K/Akt pathway, and shows an inverse pattern of expression to VEGF and EPO [47]. Intravitreal bevacizumab injections have been found to reduce aqueous VEGF and increase PEDF in patients with choroidal neovascularization secondary to age-related macular degeneration or pathologic myopia [48].

Insulin-like growth factor (IGF)-1 and its receptor, IGF-1R, have also been implicated in choroidal neovascularization. IGF-1 participates in angiogenesis of the developing retina in newborns [49]. Although it failed to enhance endothelial tube formation in vitro [50], it increased VEGF secretion in cultured ARPE-19 cells [51]. Accordingly, IGF-1R inhibitors may be useful tools in the treatment of conditions associated with neovascularization.

The proangiogenic CXC chemokine macrophage inflammatory protein (MIP)-2 induces endothelial cell chemotaxis [52] and promotes angiogenesis in cancer conditions [53]. MIP-2 regulates the production of VEGF, and like VEGF, it mediates the regulation of corneal neovascularization caused by bacterial infection [54]. One study showed that following infection, both the corneal resident cells and infiltrating neutrophils produced MIP-2 and VEGF, and the significantly high levels of both genes paralleled the extensive corneal neovascularization seen at later stages of the disease. Anti-MIP-2 antibody treatment significantly reduced both VEGF expression and corneal neovascularization [54]. However, these findings are controversial. Others have reported that following alkali injury in mice, the injection of neutralizing anti-mouse MIP-2 antibodies had no effect on corneal neovascularization [55].

The present study was conducted in a mouse model of chemically-induced corneal neovascularization. The aim of the study was to evaluate the effect of treatment with bevacizumab by three different routes of injection (subconjuctival, intracameral, and intravitreal). We characterized the damage clinically and angiographically and measured the areas of neovascularization in vivo and on histological and flat-mount sections. Molecular analysis of changes in the levels of expression of angiogenesis-related genes was performed with real-time polymerase chain reaction (PCR). In addition, given the low affinity of bevacizumab to murine VEGF [56], we also qualitatively investigated the ocular effects of bevacizumab treatment in a rabbit model.

## Methods

### Animals

The effect of injection route of bevacizumab on corneal neovascularization following a chemical burn was investigated in 119 C57BL57 male mice aged 6–8 weeks and weighing 20–25 g (Harlan Laboratories, Jerusalem). In addition, the qualitative effect of treatment was studied in six female outbreed commercial rabbits (Kfar HaNagid, Israel) weighing 2–2.5 kg and aged six months.

All protocols were conducted in accordance with the ARVO Statement for the Use of Animals in Ophthalmic and Vision Research and were approved and monitored by the Animal Care Committee of Rabin Medical Center. The animals were housed under a 14 h:10 h light-dark cycle with standard chow and water ad libitum.

### Induction of corneal neovascularization

#### Mouse model

The mice were placed under general anesthesia by intramuscular injection of combined ketamine/xylazine (80 mg/kg and 8 mg/kg, respectively) supplemented by topical anesthesia (proparacaine hydrochloride 0.5%). The silver nitrate cauterization technique described by Mahoney and Waterbury [57] was used to induce corneal neovascularization. Briefly, under the operating microscope, an applicator stick coated with 75% silver nitrate and 25% potassium nitrate with a diameter of 1.8 mm was pressed on the central right cornea of each animal for 10 seconds. Excess chemical reagent was removed by rinsing the eyes with 5 ml of balanced salt solution (NaCl 0.9%). The left eyes were not injured and served as controls. To increase the reproducibility of the injuries, a single investigator (N.G.-C.) cauterized all animals.

#### Rabbit model

A similar technique was used to induce central chemical burn in the right eye of the rabbits.

### Bevacizumab injections

#### Mouse model

Immediately after cauterization, the treated mice (n=63, Table 1) received a single injection of bevacizumab (25 mg/ml) into the right eye via a Hamilton syringe with a 30-gauge tip (Hamilton, Reno, NV). Injections were delivered by one of three routes: subconjuctival: 2.5 mg/0.1 ml (n= 22), intravitreal: 0.75 mg/0.03 ml (n= 20), or intracameral: 0.5 mg/0.02 ml (n= 21). The left eyes were untreated.

**Table 1:** Mice used in the study.

**Procedure**	**Model-untreated** **(n=56)**	**Bevacizumab-treated (n=63)**			
		**SC (n=22)**	**IV (n=20)**	**IC (n=21)**	**Total (n=63)**
In vivo digital photographs	32	20	18	19	57
India ink (flat cornea)	2	2	2	2	6
Fluorescein angiography	4	-	-	-	-
Histology (H&E)	8	8	8	8	24
Corneal flat mounts	4	-	-	-	-
Antibody staining	-	-	4	-	4
Real-time PCR	10	12	12	19	57

Several mice underwent more than one procedure. Abbreviations are: SC-subconjunctival, IV-intravitreal, IC-intracameral.

#### Rabbit model

Bevacizumab was injected into the anterior chamber immediately after induction of chemical burn to the rabbit cornea (n=6).

### Digital photographs

To quantify the extent of neovascularization, photographs were taken at 2, 4, 8, 10 and 14 days after cauterization in the mice and rabbits with a Canon A610 digital camera attached to a slit-lamp microscope. The corneal surface area containing neovascular vessels was measured on the photographs as the percentage of the total area of the cornea. Image analysis was performed on each cornea using a public domain Java image processing program developed at the National Institutes of Health, Washington, D.C.

### India ink perfusion

Mice (n=8; six treated, two untreated) were anesthetized with ketamine/xylazine on day 10 after corneal cauterization and perfused with 1 ml of waterproof Sanford-Higgins Black India Ink for drawing (Sanford, Oakbrook, IL). The eyes were removed and the corneas (with limbal regions) were isolated, radial-cut and flat-mounted. The samples were reviewed under a light microscope for new vessels and photographed.

### Fluorescein angiography

To confirm the growth of new pathological vessels, untreated mice (n=4) were anesthetized for fluorescein angiography on day 10 after corneal cauterization. Animals were injected with 0.04 ml of 25% sodium fluorescein (AK-Fluor 25% AMP; Akorn, Decatur, IL), and the procedure was performed using a digital fundus camera (Topcon TRC 50x, Farmingdale, NY). Thereafter, the mice were euthanized, and flat corneas filled with the fluorescein dye were analyzed under the microscope.

### Histological study

After euthanization of both the treated and untreated groups (n=24, Table 1), both eyes from each subject were enucleated and embedded in paraffin, and 5-μm thick sagittal sections were stained with hematoxylin and eosin and examined under a light microscope. The disruption of the corneal structure and the presence of neovascularization were documented. Measurements were performed in triplicate at 20 μm intervals at all corneal thicknesses.

### Corneal flat-mount immunostaining

Mice (n=4) were euthanized by CO_2_ inhalation on day 8 after cauterization to study the endothelium. The eyes were enucleated and the corneas isolated and fixed in 4% formaldehyde. Immunochemistry staining for the endothelial marker was performed to detect pathological blood vessels in the cornea. Briefly, corneal flat-mounts were rinsed several times in phosphate buffered saline, blocked with 1% bovine serum albumin, stained with Rat Anti-Mouse Pecam-1 [CD31] monoclonal antibody overnight (1:100; CBL1337, Chemicon, Bellirica, MA), washed three times for 5 min with PBS and stained with goat anti-rat IgG-1 for 1 h at room temperature (1:200; A11077, Molecular Probes, Eugene, OR).

### Antibody staining for intraocular bevacizumab

Eyes (n=4) were enucleated 1 day after intravitreal injection, fixed in formalin, embedded in paraffin wax, sectioned and deparaffinized. Donkey anti-human Cy3-labeled IgG (dilution 1:100; 69901, Jackson ImmunoResearch Laboratories, West Grove, PA) was used for bevacizumab detection. This polyclonal antibody binds to epitopes of both Fc and Fab portions of human IgG. Untreated control eyes were stained in the same way. The sections were then examined with a confocal microscope (Zeiss 510).

### Molecular analysis

Corneal tissue was dissected and snap-frozen in liquid nitrogen. Total RNA was isolated using a TRIzol^TM^ reagent (Invitrogen, Life Technologies, Carlsbad, CA), according to the manufacturer’s protocol, and then reverse-transcribed into cDNA using random hexamers (Bioline, London, UK) and Moloney murine leukemia virus (M-MLV)-reverse transcriptase (Promega, Madison, WI).

Two-stage real-time quantitative PCR (Sequence Detection System, Prism 7900; Applied Biosystems, Inc., Foster City, CA) was applied to evaluate the genes encoding *VEGF*, *IGF-1*, *MIP-2*, *PEDF*, and beta actin (*ACTB*) after cauterization (for primer list, see Table 2). Mouse *β-actin* was used to normalize the cDNA input levels. Reactions were performed in a 20 µl volume containing 4 µl cDNA, 0.5 µM each of forward and reverse primers and buffer included in the master mix (SYBR^®^ Green I; Applied Biosystems, Inc.). Duplicate reactions were performed for each gene to minimize individual tube variability, and an average was taken for each time point. Threshold cycle efficiency corrections were calculated, and melting curves were obtained using cDNA for each individual gene PCR assay.

**Table 2:** List of primers.

**Gene**	**Forward (5’-3’)**	**Reverse (3’-5’)**
*VEGF*	CACGACAGAAGGAGAGCAGAA	CGCTGGTAGACGTCCATGA
*MIP-2*	CTGTATTCCCCTCCATCGTG	CTCGTCACCCACATAGGAGTG
*IGF-1*	AGAGACCCTTTGCGGGGC	CGGATAGAGCGGGCTGCTT
*PEDF*	GGACTCTGATCTCAACTGCAAGA	GGAGGAAGAAGATGATGCTCATAC
*ACTB*	TAGGCACCAGGGTGTGATGGT	CATGTCGTCCCAGTTGGTAACA

PCR cycling conditions consisted of an initial denaturation step of 95 °C for 10 min followed by 40 cycles of 15 s denaturation at 95 °C and 1 min of annealing and extension at 60 °C. Standard curves were obtained using untreated mouse cDNA for each gene PCR assay. The results were quantified using a comparative threshold cycle (Ct) method, also known as the 2^-ΔΔCt^ method [58], where: ΔΔCt = ΔCt(sample) – ΔCt (reference gene).

For each treatment, the levels of expression in the cauterized right eye were compared to the untreated eye, which served as an internal control.

### Statistical analysis

Differences between groups were analyzed using an unpaired Student t-test. Significance was set at p<0.05.

## Results

### Area of neovascularization

#### Mouse model

The mean burn area in the center of the cornea measured 10% of the total corneal area in the untreated mice. In all mice subjected to chemical burn, corneal neovascularization started on day 2 and reached its maximum on days 8–10. We calculated the relative area of corneal neovascularization as 11.24% (±7.0) on day 2, 19.70% (±8.9) on day 4, 47.42% (±25.4) on day 8 and 50.62% (±24.7) on day 10. Spontaneous regression of the neovascularization was detected on day 14, when the calculated area of neovascularization was 26.98% (±19.9) of the corneal area (Table 3).

**Table 3:** Relative area of neovascularization (in vivo measurement by ImageJ).

**Time** **(days)**	**Model of corneal neovascularization** **(n=32)**	**Bevacizumab treated (n=57)**			
	**No treatment**	**Total (n=57)**	**Intraocular injection (n=37)**		**Sub-conjuctival** **injection** **(n=20)**
			**Intra-cameral** **(n=19)**	**Intra-vitreal** **(n=18)**	
2	11.24±7.02	10.98±8.79 p=0.88	14.41±2.98 p=0.06	11.05±2.49 p=0.91	7.05±2.88 *p=0.012
4	19.70±8.96	14.76±8.79 *p=0.01	16.51±6.27 p=0.18	14.68±5.05 p=0.03	15.69±6.00 p=0.09
8	47.42±25.45	27.10±21.84 *p=0.0001	26.28±6.53 *p=0.005	23.51±6.51 *p=0.001	32.16±15.21 p=0.04
10	50.62±24.74	28.29±20.92 *p=0.001	19.86±1.23 *p=0.0004	24.20±14.87 p=0.02	39.73±14.51 P=0.27
14	26.98±19.90	10.97±10.84 *p=0.002	5.28±5.90 *p=0.01	4.16±6.43 *p=0.009	23.48±17.32 p=0.68

The asterisk indicates a p<0.05.

In the bevacizumab-treated mice, the relative area of neovascularization in the corneas was lower at all time points than in the untreated, cauterized mice (Table 3). Differences between the groups were significant on days 8, 10 and 14 (p<0.005).

#### Qualitative rabbit model

**Figure 1:**
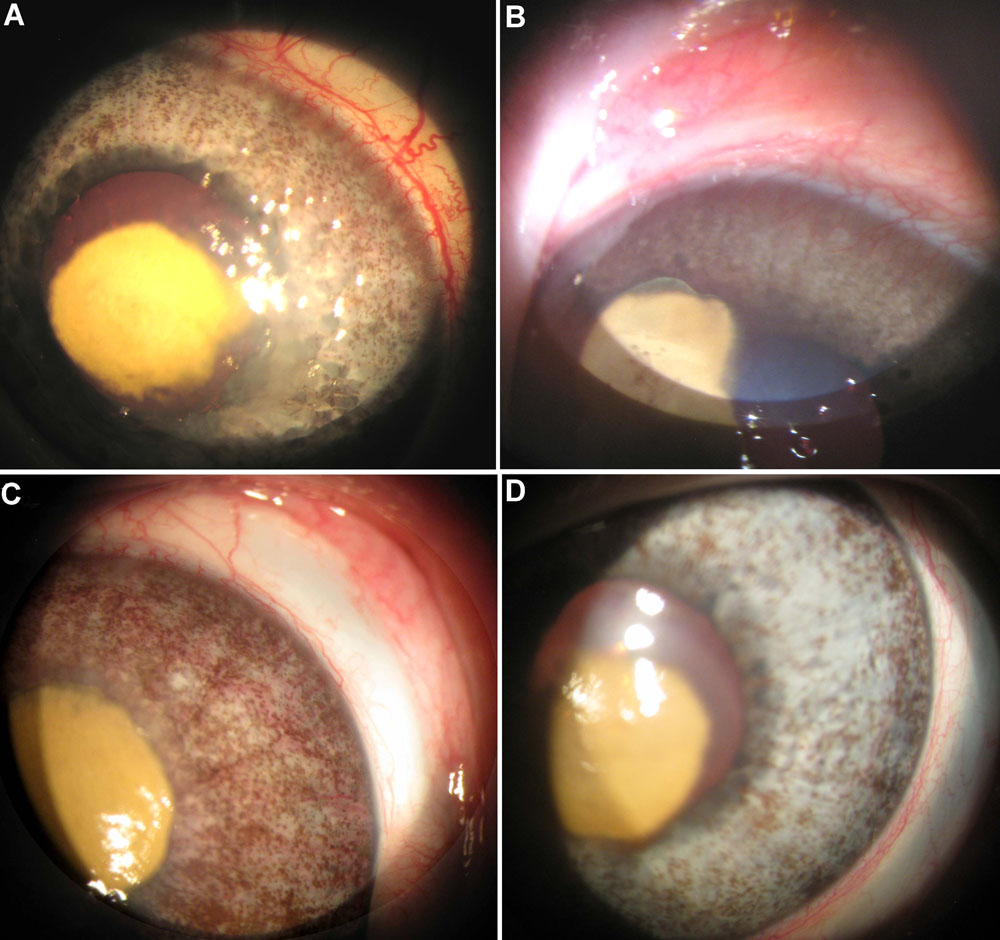
Corneal neovascularization in rabbits. **A**-**B**: Clinical appearance of neovascularization in a rabbit cornea 10 days (peak) after chemical burn induction. Note the central scar (yellow) covering approximately 10% of the corneal area. **C**-**D**: Intracameral bevacizumab-treated cornea at 10 days. Note the reduced neovascularization.

In the rabbits (n=6), the mean burn area measured 10% of the corneal area of the untreated eyes. Neovascularization was induced by the chemical burn in all rabbits starting on day 2 and achieved its maximum on day 8. Neovascularization covered less than 20% of the corneas at sacrifice (Figure 1).

The intracameral (anterior chamber) injection of bevacizumab reduced the neovascularization in the rabbits. Moreover, the limbal congestion noted in the untreated model did not develop.

### Route of injection

**Figure 2:**
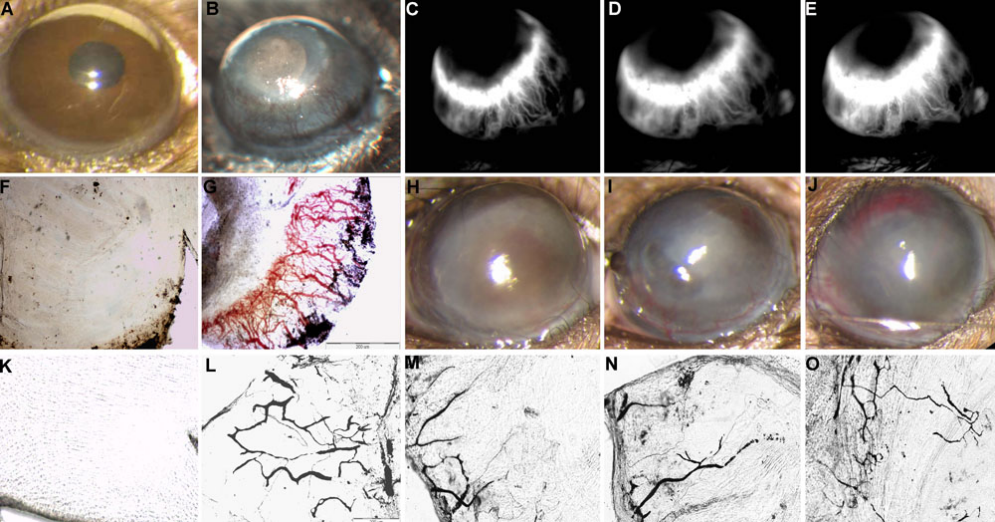
Clinical appearance of neovascularization in a mouse cornea. Comparison between normal eye, without blood vessels on the cornea (**A**) and 8 days following cauterization without treatment, with high level of neovascularization (**B**). Less blood vessels observed after bevacizumab treatment by (**H**) intravitreal injection, (**I**) intracameral injection or (**J**) subconjunctival injection at the same time point. Fluorescein angiography 8 days after chemical burn induction showing (**C**) early leakage and (**D-E**) increased late leakage. Flat mount cornea of the control mouse showing transparent cornea without blood vessels (**F**). In the neovascular corneal model, studies with fluorescein dye (**G**) and India ink (**K**, black) reveal new vessels 8 days after induction of chemical burn, compared to the control avascular cornea (**K**), and a reduced level of NV detected following bevacizumab treatment (**M-O**).

All three injection routes of bevacizumab — intravitreal, intracameral and subconjuctival — reduced growth of new abnormal vessels in the mice (Figure 2H-J). However, intraocular injections were the most effective (p<0.005, 8 and 10 days after injury).

### Fluorescein angiography

In the neovascular model, following an intraperitoneal injection of fluorescein, angiography demonstrated new vessels in the cornea on day 8 (maximum growth). The vessels were located at 360 degrees along the periphery cornea, arising from the limbus and extending towards the central scar. The new vessels showed early leakage that increased with time (Figure 2C-E).

Immediately following fluorescein angiography, the eyes were enucleated for flat-mount studies to validate the area of neovascularization (Figure 2F-G). Intraluminal fluorescein dye analysis demonstrated peripheral neovascularization of about 50% of the corneal area at 8 days after cauterization (Figure 2G).

### India ink perfusion

Flat-mount corneal studies following perfusion with India ink were conducted 10 days after cauterization. Patent vessels were noted in the corneal periphery covering 50% of the corneal area (Figure 2L). After bevacizumab treatment, the area of neovascularization was significantly reduced (Figure 2M-O).

### Histology

**Figure 3:**
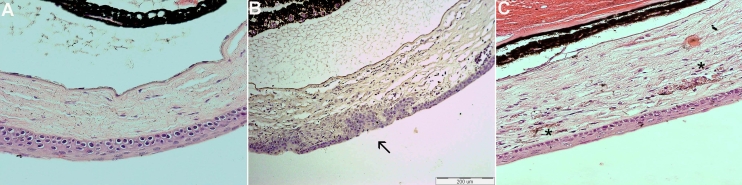
Histology analysis using hematoxylin-eosin staining. No blood vessels detected in the normal cornea (**A**). Following cauterization, development of the scar (arrow) was observed in the epithelial and anterior stroma of the center cornea (**B**). New pathological blood vessels (**C**) were located in the superficial stroma, filled with erythrocytes (*).

Histological sections stained with hematoxylin and eosin showed scarring in the epithelial and anterior stroma of the central corena after cauterization. New pathological blood vessels were located in the superficial stroma, filled with erythrocytes (Figure 3). Neovascularization was detected in both untreated and treated corneas.

### Staining for endothelial marker

**Figure 4:**
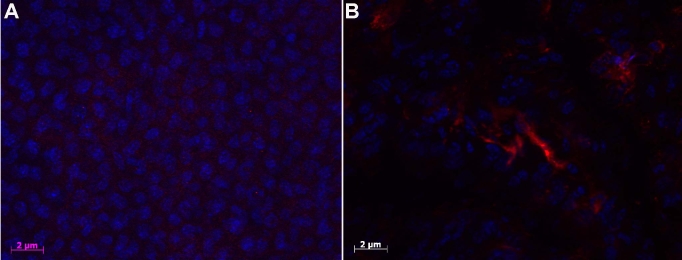
Corneal flat-mount immunostaining for endothelial marker. Flat-mount corneas were stained with endothelial marker using anti-CD31 antibody. No positive staining detected in a control mouse (avascular cornea; **A**) compared to blood vessels detected in the flat mount cornea  8 days after cauterization (red staining; **B**).

Blood vessels in the flat-mount corneas were stained with CD31, as shown in Figure 4. Staining was negative for endothelial marker in the control avascular cornea.

### Staining for intraocular bevacizumab

**Figure 5:**
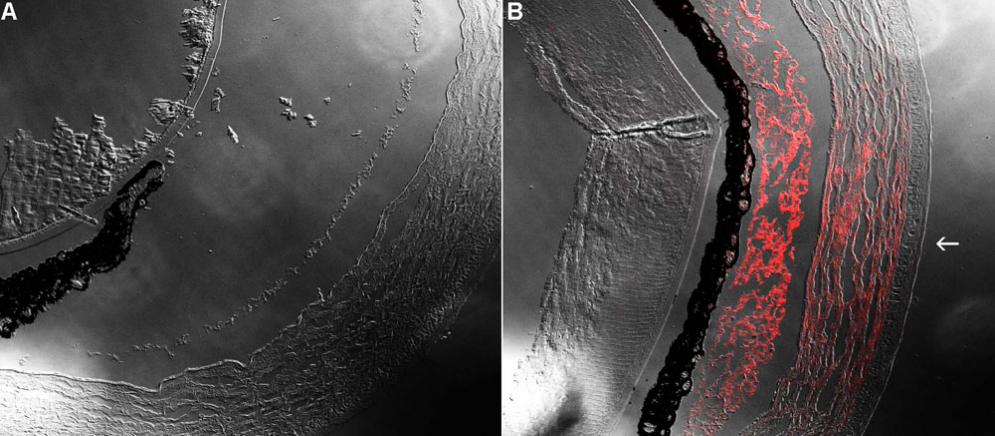
Immunostaining for intraocular bevacizumab. No staining was detected using Anti-human antibody labeled with CY3 dye in an untreated mouse after chemical burn injury (**A**) without bevacizumab injection. Bevacizumab staining identified in the anterior chamber, filtrating into the cornea, following intraocular injection one day after injury (**B**).

One day after intravitreal injection of 0.75 mg in 0.03 ml of bevacizumab, staining with anti-human Cy3TM conjugated affine pure fragment donkey anti-human IgG revealed bevacizumab in the anterior chamber, filtrating into the cornea (Figure 5B). No such findings were detected in the negative controls (cauterization without bevacizumab treatment; Figure 5A).

### Molecular analysis

#### Vascular Endothelial Growth Factor

**Figure 6:**
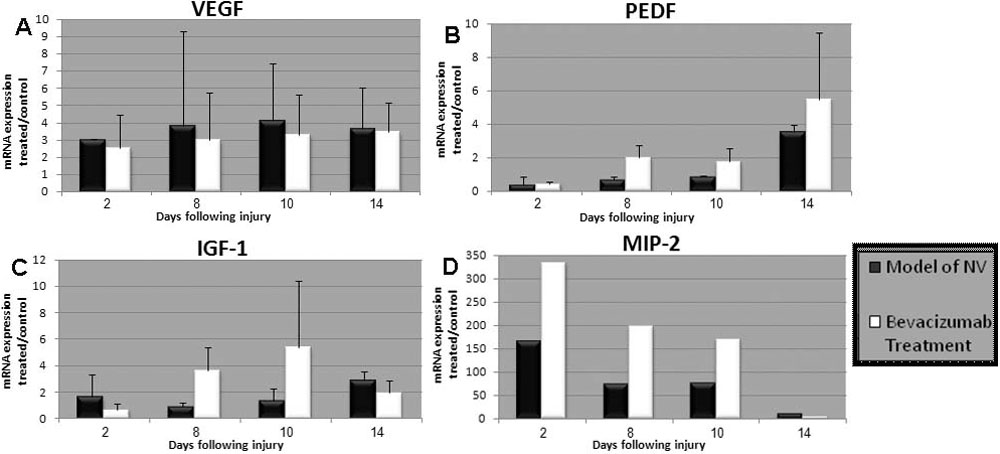
Molecular analysis of gene expression levels after chemical cauterization in the untreated and bevacizumab treated mice. *VEGF* expression up-regulated in all time points, with a slight decrease following bevacizumab treatment (**A**). *PEDF* showed an inverse pattern of expression to *VEGF* (**B**). *IGF-1* expression increased between days 8 and 10 following injury, with a higher level in the bevacizumab treated group (**C**). *MIP-2* expression significantly increased in both the untreated and treated mice on day 2 and then dropped to near-normal levels by day 14, with higher level in the bevacizumab treated eyes (**D**).

In the untreated eyes, VEGF expression peaked on day 10 and decreased slightly on day 14, in line with the clinical observations. The bevacizumab-treated eyes showed a slight increase in VEGF expression from day 2 (2.49 fold) to day 14 (3.43 fold) (Table 4, Figure 6A).

**Table 4:** Gene expression in the untreated and bevacizumab-treated corneas.

**Time point** **Gene**	**2 days**		**8 days**		**10 days**		**14 days**	
	**Model**	**Treated**	**Model**	**Treated**	**Model**	**Treated**	**Model**	**Treated**
*VEGF*	2.98 (±0.04)	2.49 (±1.95)	3.80 (±5.50)	2.96 (±2.78)	4.10 (±3.30)	3.27 (±2.32)	3.64 (±2.41)	3.43 (±1.71)
*PEDF*	0.35 (±0.48)	0.44 (±0.09)	0.65 (±0.19)	2.0 (±0.7)	0.84 (±0.08)	1.78 (±0.78)	3.54 (±0.37)	5.48 (±3.97)
*IGF-1*	1.65 (±1.65)	0.66 (±0.45)	0.90 (±0.31)	3.60 (±1.76)	1.37 (±0.85)	5.41 (±4.96)	2.88 (±0.68)	1.94 (±0.89)
*MIP-2*	166.43	332.53	75.25	197.11	76.25	170.83	11.45	4.63

#### Pigment Epithelium Derived Factor

PEDF showed an inverse pattern of expression to VEGF. After untreated cauterization, PEDF gene expression was 0.35 on day 2, with a mild increase toward baseline until day 10; by day 14, expression measured 3.54 fold. With bevacizumab treatment, PEDF levels decreased on day 2 (0.44 fold), followed by a 2-fold increase on days 8 and 10, and a 5.48-fold increase on day 14 (Table 4, Figure 6B).

#### Insulin-Like Growth Factor-1

In the untreated eyes, IGF-I expression increased on day 2, returned to baseline on day 8 and increased again on day 14 (2.88 fold). With bevacizumab treatment, levels decreased on day 2, significantly increased on day 8 (3.6 fold), peaked on day 10 (5.4-fold) and decreased on day 14 (1.94 fold; Table 4, Figure 6C).

#### Macrophage-Inflammatory Protein-2

MIP-2 expression significantly increased in both the untreated and treated mice on day 2 and then dropped to near-normal levels by day 14. However, in the treated group, the levels increased to over 300 fold, followed by a reduction to 200 fold. In the untreated group, the maximal level achieved was only 166 fold (Table 4, Figure 6D).

## Discussion

The avascular, transparent nature of the cornea makes it possible for researchers to detect and measure new blood vessel development [59]. The present study molecularly and histologically characterized a mice model of corneal neovascularization and measured the effect of bevacizumab treatment on the appearance of new vessels and the changes in angiogenesis-related genes using three different routes of injection: intravitreal, intracameral or subconjuctival.

We found that neovascularization appeared two days after the chemical burn was induced, which then progressed from the limbal vascular plexus toward the cauterization site in the central cornea. Maximal vessel growth was observed 8–10 days after injury, with spontaneous regression thereafter. These findings are in agreement with other reports of corneal neovascularization in mouse [3], rat [60], rabbit [17] and guinea pig [61] models. On day 10, the maximal area of neovascularization in our study measured 51% of the corneal area, compared to 23.5% in the guinea pig after 10 days [61], and 72% [34], 68% [60] and 63% [20] in rats after one week. The reason for the lower rate in the guinea pig is unclear, though it is probably unrelated to the different corneal size.

In vivo analysis of our model with fluorescein angiography demonstrated early leakage. Analysis with India ink showed the complex of the vessel tree in the flat-mount cornea. Histologically, the vessels were located in the superficial stroma, resembling the pathology described in human corneas following keratoplasty [62].

A single injection of bevacizumab reduced the corneal neovascularization relative to the untreated, injured mouse model. The effect was greater with the intraocular route (anterior chamber or vitreous) than with the subconjuctival route. The effect of a subconjuctival bevacizumab injection has been previously investigated in rat [63], guinea pig [61] and rabbit models [21,43,64] and in humans [41]. Hurmeric et al. [61] noted a significant reduction in corneal neovascularization when bevacizumab was injected subconjuctivally immediately after cauterization and again on day 3 after injury. Delayed injections (days 3 and 5) had a significantly weaker effect. The adjuvant injection as well as species differences may account for the superior results of this study compared to ours. In an earlier rabbit model [43], subconjunctival injection of bevacizumab caused significant inhibition of corneal neovascularization.

The intravitreal route is commonly used nowadays, with only minor complications, in patients who require repeated injections for age-related macular degeneration [46], Best disease [65] or macular edema of central retinal vein occlusion [66]. In the present study, intravitreal injection of bevacizumab was associated with a 50% reduction in corneal neovascularization compared to the untreated model (p<0.001; Figure 2). There were no injection-related complications. Surprisingly, intravitreal injection had a better effect on regression of the corneal neovascularization than injections into the anterior chamber, which come into contact with the cornea. However, the difference was not statistically significant. Immunostaining was performed to better understand the location of the intravitreally-injected bevacizumab, and it revealed that the agent was in the anterior chamber and penetrating the cornea as early as day 2 (Figure 5). Accordingly, others reported that ranibizumab (Lucentis) could be detected in the aqueous humor three days after intravitreal administration [67]. These authors attributed the drug’s effectiveness to the shift from the vitreous to the aqueous humor, and possibly to the presence of the drug in the anterior chamber one day after induction of injury [67]. We assumed that in our study, bevacizumab reached the corneal stroma by passive diffusion. Chen et al. [43] reported positive staining for bevacizumab in the corneal stroma between 3-14 days following injury induction in several different rabbit models of corneal neovascularization. They suggested that bevacizumab may be useful in preventing corneal neovascularization in the acute phase of various kinds of corneal inflammation. In a study of the effectiveness of bevacizumab in a rabbit model of retinal detachment, Dib et al. [68] detected subretinal drug molecules 2 h after intravitreal injection. Given the absence of any retinal holes or tears, they too attributed their findings to a mechanism of diffusion throughout the retinal layers.

Bevacizumab only partially inhibited neovascularization in our mouse model compared to its almost 90% inhibition of choroidal neovascularization in human eyes. The difference might be species-related, as rat, rabbit and guinea pig models all showed a better response to treatment. Yu et al. [41] found bevacizumab to be species-specific, with weak interaction with murine VEGF relative to human VEGF due to differences in the spatial molecular structure.

Although our qualitative analysis yielded a better response to bevacizumab in rabbits than in mice, we nevertheless found a consistent trend of a decrease in neovascularization following bevacizumab injection by different routes. In the wake of advances in the treatment of eye disease involving pathologic angiogenesis, interest has been directed to developing reliable and reproducible animal models [69]. Models of anterior segment neovascularization include the corneal micropocket assay, which is used to study the influence of specific molecules/proteins in angiogenesis, and suture-induced or corneal chemical injury, as in our study, which more closely mimics the complex nature of human disease [69]. The mouse model of corneal neovascularization is well-recognized as a reliable and valid model of all diseases that involve neovascularization [69]. The cornea is also well-visualized and easily accessible for qualitative and quantitative measurement. Accordingly, we were able to characterize the relative changes in the angiogenesis-related genes and to identify new aspects of the mechanisms underlying corneal neovascularization in the mice.

VEGF is required for angiogenesis [70]. In our model, there was an upregulation of VEGF mRNA expression in both the untreated and bevacizumab-treated mice, although there was slightly less in the treated group (Table 4). Yan et al. [71] conducted an in vivo study of VEGF and TSP2 expression after corneal alkali burn. They found that the level of VEGF increased at 6 h after injury and reached its maximum at 12 h. The level then increased again to additional peaks at 96 h and 192 h. The VEGF-positive reaction was concentrated mainly in the corneal stroma. This is in accordance with our detection of bevacizumab in the corneal stroma using immunohistochemical staining (Figure 5). In contrast, the expression of PEDF in the untreated mice decreased until the late stages of angiogenesis, and peaked only at 14 days following cauterization during the process of wound healing (Table 4). In the treated mice, the expression of PEDF decreased early and then increased progressively until day 14. These findings reflect the complicated interaction, with many factors involved in the promotion and inhibition of vascularization in vivo [72-74].

IGF-1 is known to induce angiogenesis [75] and may explain the upregulation at the early state of neovascularization process. In our model, IGF-1 was upregulated early in the untreated mice, with higher levels in the bevacizumab-treated group than at all the other later points. A previous study that used an ischemic mouse model also reported an increase in IGF-1 three days following injury [76]. Eight days after cauterization, there was a slight change in IGF-1 expression in the untreated mice, compared to a 3.6-fold increase in the bevacizumab-treated group. We assume that when VEGF was neutralized by bevacizumab, other proangiogenic factors, such as IGF-1, came into play.

MIP-2 is the murine analog of IL-8, a member of the proinflammatory C-X-C family of cytokines that activates the release of neutrophils during inflammation. IL-8 also serves as a proangiogenic factor during corneal neovascularization.  Similarly to IGF-1, we found an immediate, early increase in MIP-2 mRNA expression after chemical injury, which was greater in the bevacizumab-treated than in the untreated group. This finding may also be a result of the inhibition of VEGF. This finding is consistent with other studies of molecular expression during angiogenesis [77-79], but is not consistent with the conclusion of Lu et al. [55]. In this study, the authors used a similar murine model of corneal neovascularization and concluded that although MIP-2 mediated neutrophil infiltration to the corneal neovascularization, it made only a minor contribution to its development. A reduction in inflammatory cell infiltration of chemically burned rat corneas following bevacizumab injection was also reported by Oh et al. [40]. However, they noted a reduction in the expression of various cytokines, namely interleukin-2 and -6 and interferon-gamma. It is noteworthy that in this study, bevacizumab treatment led to only a borderline reduction in corneal neovascularization.

In summary, bevacizumab treatment partially inhibits the progressive corneal neovascularization induced by chemical injury in a mouse model. Treatment via the intraocular route is more effective than the subconjuctival (periocular) route. The clinical findings are compatible with the angiographic and histologic findings, and are supported by molecular analysis showing changes in the expression of pro-angiogenesis genes after treatment. Further studies of the molecular mechanisms involved in corneal neovascularization are needed in the animal models used in this study and in other animal models as well. The ability to directly block target proteins or peptides or signaling pathways holds significant promise for the development of new, targeted anti-angiogenic therapies as well as for the optimization of existing anti-angiogenic drugs or polypeptides [11].

## References

[1] Cursiefen C Chen L Saint-Geniez M Hamrah P Jin Y Rashid S Pytowski B Persaud K Wu Y Streilein JW Dana R Nonvascular VEGF receptor 3 expression by corneal epithelium maintains avascularity and vision. Proc Natl Acad Sci USA 2006 103 11405 10 16849433 10.1073/pnas.0506112103 PMC1544098

[2] Cursiefen C Rummelt C Junemann A Vorwerk C Neuhuber W Kruse FE Schroedl F Absence of blood and lymphatic vessels in the developing human cornea. Cornea 2006 25 722 6 17077668 10.1097/01.ico.0000214230.21238.3d

[3] Bock F Onderka J Dietrich T Bachmann B Kruse FE Paschke M Zahn G Cursiefen C Bevacizumab as a potent inhibitor of inflammatory corneal angiogenesis and lymphangiogenesis. Invest Ophthalmol Vis Sci 2007 48 2545 52 17525183 10.1167/iovs.06-0570

[4] Samolov B Steen B Seregard S van der Ploeg I Montan P Kvanta A Delayed inflammation-associated corneal neovascularization in MMP-2-deficient mice. Exp Eye Res 2005 80 159 66 15670794 10.1016/j.exer.2004.08.023

[5] Cursiefen C Maruyama K Jackson DG Streilein JW Kruse FE Time course of angiogenesis and lymphangiogenesis after brief corneal inflammation. Cornea 2006 25 443 7 16670483 10.1097/01.ico.0000183485.85636.ff

[6] Chang JH Gabison EE Kato T Azar DT Corneal neovascularization. Curr Opin Ophthalmol 2001 12 242 9 11507336 10.1097/00055735-200108000-00002

[7] Lee P Wang CC Adamis AP Ocular neovascularization: an epidemiologic review. Surv Ophthalmol 1998 43 245 69 9862312 10.1016/s0039-6257(98)00035-6

[8] Hashemian MN Moghimi S Kiumehr S Riazi M Amoli FA Prevention and treatment of corneal neovascularization: comparison of different doses of subconjunctival bevacizumab with corticosteriod in experimental rats. Ophthalmic Res 2009 42 90 5 19546599 10.1159/000224783

[9] Klebe S Coster DJ Williams KA Rejection and acceptance of corneal allografts. Curr Opin Organ Transplant 2009 14 4 9 19337139 10.1097/MOT.0b013e32831af1d7

[10] Lafleur MA Handsley MM Edwards DR Metalloproteinases and their inhibitors in angiogenesis. Expert Rev Mol Med 2003 5 1 39 14585170 10.1017/S1462399403006628

[11] Segal E Satchi-Fainaro R Design and development of polymer conjugates as anti-angiogenic agents. Adv Drug Deliv Rev 2009 19699248 10.1016/j.addr.2009.06.005

[12] Metro G Cappuzzo F Emerging drugs for small-cell lung cancer. Expert Opin Emerg Drugs 2009 19694501 10.1517/14728210903206983

[13] Hammady T Rabanel JM Dhanikula RS Leclair G Hildgen P Functionalized nanospheres loaded with anti-angiogenic drugs: Cellular uptake and angiosuppressive efficacy. Eur J Pharm Biopharm 2009 72 418 2719462478 19462478 10.1016/j.ejpb.2009.01.007

[14] Ferrara N Kerbel RS Angiogenesis as a therapeutic target. Nature 2005 438 967 74 16355214 10.1038/nature04483

[15] Penn JS Madan A Caldwell RB Bartoli M Caldwell RW Hartnett ME Vascular endothelial growth factor in eye disease. Prog Retin Eye Res 2008 27 331 71 18653375 10.1016/j.preteyeres.2008.05.001 PMC3682685

[16] Phillips GD Stone AM Jones BD Schultz JC Whitehead RA Knighton DR Vascular endothelial growth factor (rhVEGF165) stimulates direct angiogenesis in the rabbit cornea. In Vivo 1994 8 961 5 7539637

[17] Gan L Fagerholm P Palmblad J Vascular endothelial growth factor (VEGF) and its receptor VEGFR-2 in the regulation of corneal neovascularization and wound healing. Acta Ophthalmol Scand 2004 82 557 63 15453853 10.1111/j.1600-0420.2004.00312.x

[18] Philipp W Speicher L Humpel C Expression of vascular endothelial growth factor and its receptors in inflamed and vascularized human corneas. Invest Ophthalmol Vis Sci 2000 41 2514 22 10937562

[19] Iwasaki J Nihira S Anti-angiogenic therapy against gastrointestinal tract cancers. Jpn J Clin Oncol 2009 39 543 51 19531544 10.1093/jjco/hyp062

[20] Manzano RP Peyman GA Khan P Carvounis PE Kivilcim M Ren M Lake JC Chevez-Barrios P Inhibition of experimental corneal neovascularisation by bevacizumab (Avastin). Br J Ophthalmol 2007 91 804 7 17179168 10.1136/bjo.2006.107912 PMC1955569

[21] Papathanassiou M Theodossiadis PG Liarakos VS Rouvas A Giamarellos-Bourboulis EJ Vergados IA Inhibition of corneal neovascularization by subconjunctival bevacizumab in an animal model. Am J Ophthalmol 2008 145 424 31 18207123 10.1016/j.ajo.2007.11.003

[22] Eskens FA Verweij J The clinical toxicity profile of vascular endothelial growth factor (VEGF) and vascular endothelial growth factor receptor (VEGFR) targeting angiogenesis inhibitors; a review. Eur J Cancer 2006 42 3127 39 17098419 10.1016/j.ejca.2006.09.015

[23] Bock F Onderka J Rummelt C Dietrich T Bachmann B Kruse FE Schlotzer-Schrehardt U Cursiefen C Safety profile of topical VEGF neutralization at the cornea. Invest Ophthalmol Vis Sci 2009 50 2095 102 19151400 10.1167/iovs.07-1129

[24] Ferrara N The role of VEGF in the regulation of physiological and pathological angiogenesis. EXS 2005 94 209 31 15617481 10.1007/3-7643-7311-3_15

[25] Ferrara N Vascular endothelial growth factor: basic science and clinical progress. Endocr Rev 2004 25 581 611 15294883 10.1210/er.2003-0027

[26] Ferrara N Hillan KJ Gerber HP Novotny W Discovery and development of bevacizumab, an anti-VEGF antibody for treating cancer. Nat Rev Drug Discov 2004 3 391 400 15136787 10.1038/nrd1381

[27] Presta LG Chen H O'Connor SJ Chisholm V Meng YG Krummen L Winkler M Ferrara N Humanization of an anti-vascular endothelial growth factor monoclonal antibody for the therapy of solid tumors and other disorders. Cancer Res 1997 57 4593 9 9377574

[28] Barkmeier AJ Akduman L Bevacizumab (avastin) in ocular processes other than choroidal neovascularization. Ocul Immunol Inflamm 2009 17 109 17 19412873 10.1080/09273940802596534

[29] Grisanti S Ziemssen F Bevacizumab: off-label use in ophthalmology. Indian J Ophthalmol 2007 55 417 20 17951896 10.4103/0301-4738.36474 PMC2635984

[30] Wickremasinghe SS Michalova K Gilhotra J Guymer RH Harper CA Wong TY Qureshi S Acute intraocular inflammation after intravitreous injections of bevacizumab for treatment of neovascular age-related macular degeneration. Ophthalmology 2008 115 1911 5 18672291 10.1016/j.ophtha.2008.05.007

[31] DeStafeno JJ Kim T Topical bevacizumab therapy for corneal neovascularization. Arch Ophthalmol 2007 125 834 6 17562998 10.1001/archopht.125.6.834

[32] Kim SW Ha BJ Kim EK Tchah H Kim TI The effect of topical bevacizumab on corneal neovascularization. Ophthalmology 2008 115 e33 8 18439681 10.1016/j.ophtha.2008.02.013

[33] Uy HS Chan PS Ang RE Topical bevacizumab and ocular surface neovascularization in patients with stevens-johnson syndrome. Cornea 2008 27 70 3 18245970 10.1097/ICO.0b013e318158f6ad

[34] Erdurmus M Totan Y Subconjunctival bevacizumab for corneal neovascularization. Graefes Arch Clin Exp Ophthalmol 2007 245 1577 9 17458556 10.1007/s00417-007-0587-4

[35] Doctor PP Bhat PV Foster CS Subconjunctival bevacizumab for corneal neovascularization. Cornea 2008 27 992 5 18812760 10.1097/ICO.0b013e31817786ad

[36] Bahar I Kaiserman I McAllum P Rootman D Slomovic A Subconjunctival bevacizumab injection for corneal neovascularization in recurrent pterygium. Curr Eye Res 2008 33 23 8 18214740 10.1080/02713680701799101

[37] Dastjerdi MH Al-Arfaj KM Nallasamy N Hamrah P Jurkunas UV Pineda R 2nd Pavan-Langston D Dana R Topical bevacizumab in the treatment of corneal neovascularization: results of a prospective, open-label, noncomparative study. Arch Ophthalmol 2009 127 381 9 19365012 10.1001/archophthalmol.2009.18 PMC2703579

[38] Jacobs DS Lim M Carrasquillo KG Rosenthal P Bevacizumab for corneal neovascularization. Ophthalmology 2009 116 592 3 author reply 3-4 19264217 10.1016/j.ophtha.2008.10.011

[39] Mackenzie SE Tucker WR Poole TR Bevacizumab (Avastin) for corneal neovascularization--corneal light shield soaked application. Cornea 2009 28 246 7 19158579 10.1097/ICO.0b013e3181861cc9

[40] Oh JY Kim MK Shin MS Lee HJ Lee JH Wee WR The anti-inflammatory effect of subconjunctival bevacizumab on chemically burned rat corneas. Curr Eye Res 2009 34 85 91 19219678 10.1080/02713680802607740

[41] You IC Kang IS Lee SH Yoon KC Therapeutic effect of subconjunctival injection of bevacizumab in the treatment of corneal neovascularization. Acta Ophthalmol 2008 19021596 10.1111/j.1755-3768.2008.01399.x

[42] Han YS Lee JE Jung JW Lee JS Inhibitory effects of bevacizumab on angiogenesis and corneal neovascularization. Graefes Arch Clin Exp Ophthalmol 2009 247 541 8 18953554 10.1007/s00417-008-0976-3

[43] Chen WL Lin CT Lin NT Tu IH Li JW Chow LP Liu KR Hu FR Subconjunctival injection of bevacizumab (avastin) on corneal neovascularization in different rabbit models of corneal angiogenesis. Invest Ophthalmol Vis Sci 2009 50 1659 65 18997093 10.1167/iovs.08-1997

[44] Duch S Buchacra O Milla E Milla E Andreu D Tellez J Intracameral bevacizumab (Avastin) for neovascular glaucoma: a pilot study in 6 patients. J Glaucoma 2009 18 140 3 19225351 10.1097/IJG.0b013e318170a747

[45] Costagliola C Cipollone U Rinaldi M della Corte M Semeraro F Romano MR Intravitreal bevacizumab (Avastin) injection for neovascular glaucoma: a survey on 23 cases throughout 12-month follow-up. Br J Clin Pharmacol 2008 66 667 73 19032174 10.1111/j.1365-2125.2008.03278.x PMC2661982

[46] Kovacevic D Caljkusic-Mance T Misljenovic T Mikulicic M Alpeza-Dunato Z Intravitreal bevacizumab for the management of age-related macular degeneration. Coll Antropol 2008 32 Suppl 2 5 7 19137998

[47] Elayappan B, Ravinarayannan H, Sardar Pasha SP, Lee KJ, Gurunathan S. PEDF inhibits VEGF- and EPO- induced angiogenesis in retinal endothelial cells through interruption of PI3K/Akt phosphorylation. Angiogenesis 2009. 19657716 10.1007/s10456-009-9153-5

[48] Chan WM Lai TY Chan KP Li H Liu DT Lam DS Pang CP Changes in aqueous vascular endothelial growth factor and pigment epithelial-derived factor levels following intravitreal bevacizumab injections for choroidal neovascularization secondary to age-related macular degeneration or pathologic myopia. Retina 2008 28 1308 13 18728623 10.1097/IAE.0b013e31818358b2

[49] van Wijngaarden P Brereton HM Gibbins IL Coster DJ Williams KA Kinetics of strain-dependent differential gene expression in oxygen-induced retinopathy in the rat. Exp Eye Res 2007 85 508 17 17692314 10.1016/j.exer.2007.07.001

[50] Browning AC Dua HS Amoaku WM The effects of growth factors on the proliferation and in vitro angiogenesis of human macular inner choroidal endothelial cells. Br J Ophthalmol 2008 92 1003 8 18577655 10.1136/bjo.2007.127670

[51] Economou MA Wu J Vasilcanu D Rosengren L All-Ericsson C van der Ploeg I Menu E Girnita L Axelson M Larsson O Seregard S Kvanta A Inhibition of VEGF secretion and experimental choroidal neovascularization by picropodophyllin (PPP), an inhibitor of the insulin-like growth factor-1 receptor. Invest Ophthalmol Vis Sci 2008 49 2620 6 18515591 10.1167/iovs.07-0742

[52] Moldobaeva A Baek A Wagner EM MIP-2 causes differential activation of RhoA in mouse aortic versus pulmonary artery endothelial cells. Microvasc Res 2008 75 53 8 17662312 10.1016/j.mvr.2007.06.007 PMC2258091

[53] Kollmar O Scheuer C Menger MD Schilling MK Macrophage inflammatory protein-2 promotes angiogenesis, cell migration, and tumor growth in hepatic metastasis. Ann Surg Oncol 2006 13 263 75 16424980 10.1245/ASO.2006.03.096

[54] Xue ML Thakur A Willcox M Macrophage inflammatory protein-2 and vascular endothelial growth factor regulate corneal neovascularization induced by infection with Pseudomonas aeruginosa in mice. Immunol Cell Biol 2002 80 323 7 12121220 10.1046/j.1440-1711.2002.01094.x

[55] Lu P Li L Mukaida N Zhang X Alkali-induced corneal neovascularization is independent of CXCR2-mediated neutrophil infiltration. Cornea 2007 26 199 206 17251813 10.1097/01.ico.0000248385.16896.34

[56] Yu L Wu X Cheng Z Lee CV LeCouter J Campa C Fuh G Lowman H Ferrara N Interaction between bevacizumab and murine VEGF-A: a reassessment. Invest Ophthalmol Vis Sci 2008 49 522 7 18234994 10.1167/iovs.07-1175

[57] Mahoney JM Waterbury LD Drug effects on the neovascularization response to silver nitrate cauterization of the rat cornea. Curr Eye Res 1985 4 531 5 2410194 10.3109/02713688508999984

[58] Livak KJ Schmittgen TD Analysis of relative gene expression data using real-time quantitative PCR and the 2(-Delta Delta C(T)). Methods 2001 25 402 8 11846609 10.1006/meth.2001.1262

[59] Regenfuss B Bock F Parthasarathy A Cursiefen C Corneal (lymph)angiogenesis--from bedside to bench and back: a tribute to Judah Folkman. Lymphat Res Biol 2008 6 191 201 19093792 10.1089/lrb.2008.6348

[60] Peyman GA Kivilcim M Morales AM DellaCroce JT Conway MD Inhibition of corneal angiogenesis by ascorbic acid in the rat model. Graefes Arch Clin Exp Ophthalmol 2007 245 1461 7 17318569 10.1007/s00417-007-0542-4

[61] Hurmeric V Mumcuoglu T Erdurman C Kurt B Dagli O Durukan AH Effect of subconjunctival bevacizumab (Avastin) on experimental corneal neovascularization in guinea pigs. Cornea 2008 27 357 62 18362668 10.1097/ICO.0b013e318160d019

[62] Naoumidi TL Pallikaris IG Naoumidi II Astyrakakis NI Conductive keratoplasty: histological study of human corneas. Am J Ophthalmol 2005 140 984 92 16376640 10.1016/j.ajo.2005.06.027

[63] Barros LF Belfort R Jr The effects of the subconjunctival injection of bevacizumab (Avastin) on angiogenesis in the rat cornea. An Acad Bras Cienc 2007 79 389 94 17768531 10.1590/s0001-37652007000300004

[64] Kim TI Kim SW Kim S Kim T Kim EK Inhibition of experimental corneal neovascularization by using subconjunctival injection of bevacizumab (Avastin). Cornea 2008 27 349 52 18362666 10.1097/ICO.0b013e31815cf67d

[65] Cakir M Cekic O Yilmaz OF Intravitreal bevacizumab and triamcinolone treatment for choroidal neovascularization in Best disease. J AAPOS 2009 13 94 6 19101187 10.1016/j.jaapos.2008.06.014

[66] Beutel J, Ziemssen F, Luke M, Partsch M, Bartz-Schmidt KU, Gelisken F. Intravitreal bevacizumab treatment of macular edema in central retinal vein occlusion: one-year results. Int Ophthalmol 2008. 19099203 10.1007/s10792-008-9282-7

[67] Bakri SJ Snyder MR Reid JM Pulido JS Ezzat MK Singh RJ Pharmacokinetics of intravitreal ranibizumab (Lucentis). Ophthalmology 2007 114 2179 82 18054637 10.1016/j.ophtha.2007.09.012

[68] Dib E Maia M Longo-Maugeri IM Martins MC Mussalem JS Squaiella CC Penha FM Magalhaes O Jr Rodrigues EB Farah ME Subretinal bevacizumab detection after intravitreous injection in rabbits. Invest Ophthalmol Vis Sci 2008 49 1097 100 18326736 10.1167/iovs.07-1225

[69] Montezuma SR Vavvas D Miller JW Review of the ocular angiogenesis animal models. Semin Ophthalmol 2009 24 52 61 19373687 10.1080/08820530902800017

[70] Ferrara N Gerber HP LeCouter J The biology of VEGF and its receptors. Nat Med 2003 9 669 76 12778165 10.1038/nm0603-669

[71] Yan J Zeng Y Jiang J Zhou J Yin Z Wang Z Zhu P The expression patterns of vascular endothelial growth factor and thrombospondin 2 after corneal alkali burn. Colloids Surf B Biointerfaces 2007 60 105 9 17651946 10.1016/j.colsurfb.2007.06.013

[72] Tombran-Tink J Aparicio S Xu X Tink AR Lara N Sawant S Barnstable CJ Zhang SS PEDF and the serpins: phylogeny, sequence conservation, and functional domains. J Struct Biol 2005 151 130 50 16040252 10.1016/j.jsb.2005.05.005

[73] Tombran-Tink J Barnstable CJ PEDF: a multifaceted neurotrophic factor. Nat Rev Neurosci 2003 4 628 36 12894238 10.1038/nrn1176

[74] Tombran-Tink J Barnstable CJ Therapeutic prospects for PEDF: more than a promising angiogenesis inhibitor. Trends Mol Med 2003 9 244 50 12829012 10.1016/s1471-4914(03)00074-1

[75] Harper SJ Bates DO VEGF-A splicing: the key to anti-angiogenic therapeutics? Nat Rev Cancer 2008 8 880 7 18923433 10.1038/nrc2505 PMC2613352

[76] Sivakumar V Zhang Y Ling EA Foulds WS Kaur C Insulin-like growth factors, angiopoietin-2, and pigment epithelium-derived growth factor in the hypoxic retina. J Neurosci Res 2008 86 702 11 17943991 10.1002/jnr.21519

[77] Kvanta A Neovascular age-related macular degeneration: too many theories, too little knowledge? Acta Ophthalmol 2008 86 468 9 18752524 10.1111/j.1755-3768.2008.01283.x

[78] Norrby K Interleukin-8 and de novo mammalian angiogenesis. Cell Prolif 1996 29 315 23 8809123 10.1111/j.1365-2184.1996.tb01583.x

[79] Norrby K Vascular endothelial growth factor and de novo mammalian angiogenesis. Microvasc Res 1996 51 153 63 8778571 10.1006/mvre.1996.0017

